# The Use of 2-Octylcyanoacrylate Dressing in Reducing the Risk of Superficial Surgical Site Infections in Colorectal Stoma Surgery: A Retrospective Cohort Study

**DOI:** 10.7759/cureus.41708

**Published:** 2023-07-11

**Authors:** Ishtiyaq Bukhari, Zeeshan Afzal, Nicholas Judkins, Dominic M Summers, Robert Dennis

**Affiliations:** 1 Department of Surgery, Peterborough City Hospital, Peterborough, GBR; 2 Department of Surgery, University of Cambridge, Cambridge, GBR

**Keywords:** surgical site infection, stoma, skin glue, dermabond, 2-octylcyanoacrylate

## Abstract

Background: Superficial surgical site infection (SSI) is a common morbidity following bowel resection surgery involving stoma formation with clinical and financial implications. The study aimed to evaluate the role of topical skin adhesive, 2-octylcyanoacrylate (Dermabond®) (2-OCA) in reducing wound infections following colorectal stoma surgery.

Methods: We performed a retrospective, single-centre, cohort study using clinical notes. All patients, over the age of 18, undergoing bowel resection either elective or emergency, with stoma formation over five years from January 2015 to December 2019 were included. The primary endpoint was SSI, defined by the clinical manifestation of inflammation including pain, erythema, and discharge, regardless of the microbiological culture results. Patients received either 2-OCA glue as wound dressing or standard firm adhesive wound dressing e.g. Opsite.

Results: Overall, 604 patients were included in the study. The median age was 67; 187 (31%) patients received Dermabond (Group 1) and 417 (69%) received standard care (Group 2). A total of 288 (47%) patients were female, 134 (22%) had body mass index (BMI) greater than 30, 87 (14%) were diabetic, and 90 (15%) were smokers. A total of 279 (46%) patients had an American Society of Anesthesiologists (ASA) score of 3 and 4; 282 (47%) patients went through emergency surgery, 279 (64%) patients underwent dirty surgery, and 220 (35%) patients developed SSI. BMI greater than 30 compared to < 30 (OR: 2.32, 95% CI: 1.54-3.49, p<0.0001), diabetes compared to no diabetes (OR: 0.54, 95% CI: 0.32-0.92, p<0.0241), dirty surgery compared to clean surgery (OR: 2.26, 95% CI: 1.51-3.37, p<0.0001) and standard care, no 2-OCA glue use compared to the use of 2-OCA glue (OR: 1.52, 95% CI: 1.03-2.24, p=0.0343) were associated with SSIs.

Conclusion: Our study demonstrates that there is an association between 2-OCA and reduced SSIs in bowel resection surgery involving stoma formation when compared to standard methods of wound dressing. Further randomised clinical trials are recommended to strengthen this evidence and demonstrate causation.

## Introduction

Surgical site infection (SSIs) is one of the most common post-operative complications and is a significant cause of patient morbidity, contributing towards an increased expense in terms of prolonged hospital stay, antibiotic use, return to theatre, and dressing changes. There are no specific standards for SSI rate in stoma surgery; however, literature reports SSI of 18%-40% for all types of colorectal surgery [1,2]. There are several negative outcomes for patients and surgeons as well as financial implications for hospitals [3]. A multimodal approach of an ‘SSI care bundle’ has been shown to reduce the incidence of SSIs and is regarded as standard surgical practice [4]. Whilst antibiotic administration, normothermia, and skin antisepsis provide the cornerstones to such SSI care bundles, other techniques and interventions have also been proposed as potentially reducing rates of SSIs.

The use of topical skin adhesives such as 2-octylcyanoacrylate (Dermabond) (2-OCA) may be used as a method of closing wounds and as a wound dressing. 2-OCA is thought to provide a watertight wound dressing that avoids the need for any other dressings and may help protect the wound from contamination. The use of topical skin adhesive as a wound dressing was first described in open colorectal surgery in 2001 [5]. The strongest evidence to date for the use of 2-OCA to reduce SSIs comes from trials in spinal and plastic surgery where it was associated with a lower rate of SSIs as compared to other wound closure methods [6,7]. It has the potential advantages of offering watertight protection against faecal contamination in the event of leakages from the stoma appliance and avoiding overlapping wound dressings and stoma appliances. However, the small amount of subsequently published evidence for the use of topical skin adhesives in colorectal and general surgery has shown inconsistent results. Lee et al. showed a reduction in SSIs associated with the use of 2-OCA in a retrospective analysis of 492 patients undergoing elective laparoscopic colorectal surgery [8]. Fecso et al. showed an increase in SSIs associated with the use of tissue adhesive in a retrospective analysis of 1,579 patients undergoing bariatric surgery [9].

Our study aimed to evaluate the potential effect of topical skin adhesive, 2-OCA in reducing the rates of SSIs in surgery involving a bowel resection and stoma formation.

## Materials and methods

We performed a retrospective cohort study at a single district general hospital. All patients over the age of 18 who underwent emergency or elective bowel resection with stoma formation, over five years between January 2015 to December 2019, were included in the study. Patients who had a non-colorectal stoma (urostomy), and patients undergoing successful reversal of a stoma were excluded. Patients were divided into two groups: Group 1 where 2-OCA topical skin adhesive was used as a primary method of wound dressing and Group 2 with patients receiving standard wound dressing using a variety of OpsiteR adhesive dressings. The primary outcome measure of the study was the rate of SSI in the two groups. The local ethics committee did not request institutional review board (IRB) approval since this is a retrospective study without identifying information.

In both elective and emergency cases, the most common prepping agent used was chlorhexidine 2% (chlorhexidine gluconate 2%) and there was no documented use of any post-procedure wound treatment before closure of the skin.

Patients were identified via the hospital theatre logbook and then cross-checked with the clinical notes including operation notes - this helped identify if the patients received Dermabond or standard dressing. Other data for the study were retrieved from paper and electronic patient records. We recorded patient demographics including age, gender, smoking status, BMI, American Society of Anesthesiologists (ASA) grade, and diabetic status. The timing of surgery was classified according to the National Confidential Enquiry into Patient Outcome and Death (NCEPOD) classification. This was defined as immediate: life/limb/organ-saving intervention; urgent: intervention for acute onset or clinical deterioration of potentially life-threatening conditions; expedited: patient requiring early treatment where the condition is not an immediate threat to life, limb, or organ survival; elective: intervention planned or booked in advance of routine admission. The degree of surgical contamination was defined as per the Centres for Disease Control and Prevention (CDC) definitions: 1 - clean surgery; 2 - clean/contaminated; 3 - contaminated; 4 - dirty.

Clinical notes including the first outpatient follow-up (usually eight weeks) clinic letters were reviewed to identify patients who developed SSIs during the index admission or post discharge. This process was facilitated by searching laboratory results of any wound swabs resulting in the growth of any organism or presence of pus cells. However, the SSI was defined by clinical manifestation of inflammation including pain, erythema, and discharge, regardless of the microbiological culture results. In cases where SSI was diagnosed, the mainstay of treatment included the use of antibiotics, guided by culture sensitivity and drainage of the collections, if any.

Univariate analysis was performed using the chi-square test, Fisher’s exact test, and Wilcoxon rank‐sum test. Stepwise logistic regression was performed to identify factors associated with SSI using p=0.1 to select variables for inclusion in the final model. The dataset was complete.

## Results

A total of 604 patients were included in the study from January 2015 to December 2019 (Table 1). The median age was 67 (Interquartile range 55-77). Out of 604 patients, 187 (31%) patients received Dermabond (Group 1) and 417 (69%) received standard care (Group 2).

**Table 1:** Patient Demographics BMI: body mass index; NCEPOD: National Confidential Enquiry into Patient Outcome and Death; ASA: American Society of Anesthesiologists; SSI: surgical site infection.

	Total	Group 1 (Dermabond)	Group 2 (Standard care)	
Patients	604	187 (31%)	417 (69%)	
Age				
Median	67 (55-77)	64 (50-76)	69 (58-77)	
Gender				p=0.0071
Female	288 (48%)	77 (41%)	211 (51%)	
Male	316 (52%)	110 (59%)	206 (49%)	
Smoker				p=0.0782
No	514 (85%)	162 (87%)	352 (84%)	
Yes	90 (15%)	25 (13%)	65 (16%)	
Diabetes				p=0.0954
No	517 (86%)	159 (85%)	358 (86%)	
Yes	87 (14%)	28 (15%)	59 (14%)	
BMI				p=0.0836
<30	470 (78%)	145 (76%)	325 (78%)	
>30	134 (22%)	42 (24%)	92 (22%)	
Contamination				p<0.0001
1 to 2	441 (73%)	156 (83%)	285 (68%)	
3 to 4	163 (27%)	31 (17%)	132 (32%)	
NCEPOD				p<0.0001
Elective	322 (53%)	124 (66%)	198 (47%)	
Emergency	282 (47%)	63 (34%)	219 (53%)	
ASA classification				p=0.0006
1 to 2	325 (54%)	118 (63%)	207 (50%)	
3 to 4	279 (46%)	69 (37%)	210 (50%)	
SSI				p=0.0015
No	384 (64%)	134 (72%)	250 (60%)	
Yes	220 (36%)	53 (28%)	167 (40%)	

The incidence of SSI in the whole cohort was 36% (220 out of 604). This was significantly higher in Group 2 where 167 out of 417 patients developed SSI as compared to Group 1 where 53 out of 187 developed SSI (28% vs 40% p<0.0015).

There were no significant differences in age, gender, BMI, prevalence of diabetes, or smoking status between the groups. Overall, 288 (47%) patients were female (41% vs 51% p=0.0071), and 134 (22%) patients had BMI greater than 30 (23% vs 22% p=0.0836); 87 (14%) were diabetic (15% vs 14% p=0.954) and 90 (15%) patients were smokers (13% vs 16% p=0.0782).

However, we did have significant differences in SSI risk factors between the two groups. Overall, 279 patients out of 604 were categorised as ASA 3 or 4. Of this cohort, 210 patients were in Group 2 as compared to 69 in Group 1 (37% vs 50% p=0.0006). Similarly, 282 (47%) patients went through emergency surgery of which 219 were in Group 2 as compared to 63 in Group 1 (34% vs 53% p<0.0001). And overall, 279 (64%) patients underwent contaminated and dirty surgery of which 132 were in Group 2 as compared to 31 in Group 1 (17% vs 32% p<0.0001).

On multivariate logistic regression analysis, we found that the following groups were associated with SSIs: BMI greater or equal to 30 vs. less than 30 (OR: 2.32, 95% CI: 1.54-3.49, p<0.0001), diabetes vs. non diabetes (OR: 0.54, 95% CI: 0.32-0.92, p<0.0241), dirty surgery vs. non dirty surgery (OR: 2.26, 95% CI: 1.51-3.37, p<0.0001) and standard care i.e. no Dermabond use vs. Dermabond use (OR: 1.52, 95% CI: 1.03-2.24, p=0.0343) (Table 2).

**Table 2:** Multivariate Logistic Regression Analysis BMI: body mass index

	Odds Ratio	Confidence intervals	P Value
BMI >30	2.23	1.54 – 3.49	<0.0001
BMI <30	1		
Diabetes	0.54	0.32 – 0.92	0.0241
Non-Diabetes	1		
Dirty surgery	2.26	1.51-3.37	<0.0001
Non dirty surgery	1		
Non Dermabond dressing	1.52	1.03 – 2.24	0.03
Dermabond Dressing	1		

The severity of the SSIs was categorised as follows: 1) minor - no intervention required or oral antibiotics alone, 2) moderate - requiring the opening of the skin closure and/or intravenous antibiotics, 3) major - operative intervention in the theatre. In this series, we have not identified a significant pattern of more severe SSI associated with the use of topical skin adhesives.

## Discussion

Our study shows that the use of topical skin adhesive 2-OCA is associated independently with reduced incidence of SSI in bowel resection surgery involving stoma formation as compared to standard methods of wound dressing. We would assume that this is due to the 2-OCA providing a water-resistant barrier to contamination from the stoma contents in the post-operative period. Our overall incidence of SSI of 36% (220 out of 604) is consistent with the published literature for such a broad cohort of patients undergoing bowel resection surgery and underlines the significance of the problem. Our multivariate analysis also confirms that the established SSI risk factors including BMI>30, diabetes, and dirty surgery are independent; this is reassuring in terms of having captured a representative sample.

Dermabond forms a water-resistant seal on the incision, providing extra support to the skin edges due to its adherent properties. There are concerns that such wound closures could pose a risk of serious SSIs, particularly in dirty surgery. We did not come across such problems in the 187 patients who had 2-OCA including the 31 patients undergoing dirty surgery. We would always defer to the operating surgeon to judge the technique of skin closure on a case-by-case basis but our data does support that 2-OCA can be used safely in the majority of situations.

There are other significant benefits to the use of 2-OCA in wound closure for patients who have stomas. It does not interfere with the stoma apparatus or drain sites and being transparent, it provides visibility of the wound. There is evidence that it has anti-microbial properties ideal for wound dressing [10,11]. It reduces the burden on ward nurses who need not undertake repeated dressing changes; it is well tolerated by patients and aids early patient mobilisation postoperatively [5,11]. Dermabond does carry an additional cost which we estimated to be £5.93 per case [3,12], although this does not take into account the cost savings of SSI prevention.

The strengths of our study are the number of cases that were assessed, and it is the largest published study assessing 2-OCA in this population of patients. Our data comes from our day-to-day ‘real world’ practice and should therefore be relevant and easily reproducible at other hospitals.

However, as a retrospective cohort study, our data does have some significant limitations. Our results indicate that we did not have evenly matched groups in terms of SSI risk factors, although the multivariate analysis still showed that the use of 2-OCA was an independent risk factor for the development of SSIs. Secondly, wounds were assessed as part of clinical care by individual team members and so the frequency of reporting may have varied. A prospective randomised trial would be able to address these weaknesses and our study may support such a trial.

## Conclusions

SSI is common following bowel resection surgery involving the stoma. 2-OCA (Dermabond) is a skin adhesive that provides a watertight wound dressing. Our study demonstrates that the use of Dermabond reduced SSIs in bowel resection surgery involving stoma formation as compared to standard methods of wound closure.
